# Acid-Base Balance in Healthy Adults: Beneficial Effects of Bicarbonate and Sodium-Rich Mineral Water in a Randomized Controlled Trial: The BicarboWater Study

**DOI:** 10.1155/2024/3905500

**Published:** 2024-04-09

**Authors:** Katharina Mansouri, Theresa Greupner, Edda van de Flierdt, Inga Schneider, Andreas Hahn

**Affiliations:** Institute of Food Science and Human Nutrition, Leibniz University Hanover, Hanover 30159, Germany

## Abstract

**Background:**

Noncommunicable diseases (NCDs) are a global health challenge. The complex etiology of NCDs involves genetic, environmental, and lifestyle factors, including dietary habits. Chronic latent metabolic acidosis has been associated with an increased risk of NCDs. Alkalizing diets and mineral water consumption have shown promise in improving acid-base balance and potentially impacting NCDs.

**Methods:**

In this randomized controlled intervention study, the effect of drinking 1,500–2,000 mL of mineral water daily on acid-base balance was evaluated. Ninety-four healthy participants were divided into two groups: one consumed mineral water with a high bicarbonate and sodium content (HBS, *n* = 49) and the other consumed mineral water with a low bicarbonate and sodium content (LBS, *n* = 45). Changes in venous blood gas and urinary acid-base parameters were measured over a short-term (3 days) and long-term (28 days) intervention period. Potential renal acid load (PRAL) and nutrient intake were calculated at baseline and after 28 days.

**Results:**

HBS water consumption led to increased urinary pH (24-hour urine and spontaneous urine, both *p* < 0.001) and bicarbonate levels (*p* < 0.001), accompanied by reduced titratable acids (*p* < 0.001) and ammonium (*p* < 0.001), resulting in a lower renal net acid excretion (*p* < 0.001). These changes occurred in the short term and persisted until the end of the study. LBS consumption showed no significant effects on urinary pH but led to a slight decrease in bicarbonate (*p* < 0.001) and NH_4_^+^ (*p* < 0.001), resulting in a slight decrease in NAE (*p*=0.011). Blood gas changes were modest in both groups. Mineral water consumption in the HBS group altered dietary intake of sodium and chloride, contributing to changes in PRAL values.

**Conclusion:**

The study demonstrates that the consumption of mineral water high in bicarbonate and sodium (1,500 mL–2,000 mL/day) can positively influence urinary acid-base parameters and reduce NAE, suggesting potential benefits in maintaining acid-base balance without adverse effects on human health. These findings highlight the importance of mineral water composition in acid-base regulation. This trial is registered with DRKS00025341.

## 1. Introduction

Noncommunicable diseases (NCDs) are a major global health challenge, affecting millions of people worldwide. They are among the leading causes of death and disability-adjusted life years (DALYs) in the world [1, 2]. The etiology of these diseases is complex and multifaceted, involving an interplay of genetic predisposition, environmental exposures, and lifestyle factors, particularly dietary habits. Several studies have shown a significant association between chronic latent metabolic acidosis and an increased risk of developing or progressing NCDs such as osteoporosis, type 2 diabetes, and even mental disorders or cancer [3–7]. Prolonged adherence to an acid-forming diet results in a sustained disturbance of acid-base balance, leading to chronic latent acidosis. Therefore, maintaining a balanced acid-base status through dietary and lifestyle modifications may have a positive impact on health [4, 8].

In this context, dietary patterns rich in alkalizing components have demonstrated their ability to exert a favorable influence on human acid-base balance [9–12]. Furthermore, the consumption of mineral water has emerged as a promising way to improve acid-base balance [10, 13]. The bioactive components present in mineral water, which include essential minerals such as calcium, magnesium, potassium, and sodium, are known to improve the potential renal acid load (PRAL) and counteract the acidogenic components in the diet [10, 14]. Some mineral waters also contain high levels of bicarbonate, which is also a natural component of the body's bicarbonate buffering system and plays a key role in neutralizing acids and maintaining the body's acid-base balance [15, 16]. Consistent with these findings, consumption of bicarbonate-rich mineral water and a low PRAL value have been shown to exhibit beneficial effects on markers of acid-base metabolism [10].

Nevertheless, the existing body of evidence on the effect of mineral water on urinary and blood parameters related to acid-base status remains limited. Previous studies have focused on mineral waters with different bicarbonate concentrations and PRAL values or just alkalizing water. They also mainly investigated the effects of mineral water consumption over longer time periods (2–4 weeks). The present intervention study was carried out with the aim of determining the influence of two different mineral waters on acid-base balance in healthy, omnivorous adults, with comprehensive evaluations conducted over both short-term (3 days) and long-term (4 weeks) intervention periods. These waters were characterized by either a very high bicarbonate content or a very low PRAL value (HBS) or a relatively low bicarbonate content and a moderate PRAL value (LBS).

## 2. Materials and Methods

### 2.1. Study Design

This parallel-group randomized controlled intervention study was designed to compare the influence of mineral water very high in bicarbonate and high in sodium (HBS) with mineral water low in bicarbonate and sodium (LBS) on urinary and blood parameters of the acid-base-status. The study was carried out at the Institute of Food Science and Human Nutrition at Leibniz University Hanover in Germany. It was conducted between June 2021 and May 2022.

### 2.2. Subjects

Ninety-four healthy subjects, ranging in age from 30 to 65 years, were recruited from the general population of Hanover and Hildesheim. Primary inclusion factors were an omnivorous diet and a BMI within the range of 20.0 to 29.9 kg/m^2^. Subjects with chronic conditions such as manifest cardiovascular or renal diseases, as well as those under medical treatment for hypertension, were not considered for participation.

### 2.3. Ethical Approval

The study was conducted in accordance with the ethical standards described in the Declaration of Helsinki. The study protocol was approved by the Ethics Committee of the Medical Chamber of Lower Saxony (Hanover, Germany) (BO/24/2021). After being fully informed of the purpose of the study and any potential adverse effects, all participants gave informed consent. The study was registered in the German Clinical Trial Register (DRKS00025341, https://drks.de/search/en). In adherence to established protocols for the documentation of clinical trials, the CONSORT (Consolidated Standards of Reporting Trials) checklist was implemented to ensure transparency and comprehensiveness in explaining both the methodology and results of the trial (Supplementary S1).

### 2.4. Procedure and Test Products

The study consisted of a screening and a 4-week intervention phase. The intervention included an initial examination at baseline (*t*_0_) and two further examinations after 3 days (*t*_3_) and after 4 weeks of mineral water consumption (*t*_28_). Short-term effects were investigated at *t*_3_, while long-term effects were determined at *t*_28_. Participants received the bottles of the test water for the entire study period on the first day of the intervention (*t*_0_), immediately after the examinations. Alternatively, the water was delivered to their homes in advance. Participants who received the water in advance were instructed not to start drinking it until the end of the examinations on the first examination day (*t*_0_).

Participants were randomly assigned by an independent researcher to one of the two intervention groups using stratified randomization according to the covariates sex and age (in descending order). Subjects assigned to the high bicarbonate and sodium group (HBS, *n* = 49) consumed a mineral water with a very high bicarbonate content (4,368 mg/L) and a high sodium content (1,708 mg/L). Conversely, those assigned to the low bicarbonate and low sodium group (LBS, *n* = 45) consumed a mineral water with a low bicarbonate (228 mg/L) and a low sodium (8.4 mg/L) content. The composition of the test products is shown in Table 1. Over the course of the 4-week intervention, participants were instructed to consume a minimum of 1,500 mL and a maximum of 2,000 mL of the provided test water daily. Additional fluencies as tap water in its pure form, tea, coffee, mixture of juice and tap water were allowed. Besides that, subjects were advised to maintain their usual diet and maintain their physical activity level. To monitor the consumption of the test water, participants were asked to complete a daily protocol documenting the amount of water consumed daily. This protocol was used to check compliance with the study protocol and to calculate the amount of daily test water consumption after the intervention period. Moreover, compliance was checked by a question in the questionnaire at the end of the study. Participants were considered compliant if they drank 1,500 to 2,000 mL of the respective mineral water on at least half of the intervention days. In addition, consumption of less than 1 liter per day on more than 2 intervention days resulted in exclusion from the analysis.

In order to minimize the impact of circadian rhythm-related fluctuations, all appointments for participants were set at approximately the same time within a consistent morning time frame, spanning from 6:00 to 10:00 a.m. Moreover, the study period began on Monday for all participants. The examination days (*t*_0_, *t*_3_, *t*_28_) were structured as follows: After completion of the current health status questionnaire, measurements of anthropometric characteristics were performed, followed by blood sampling.

### 2.5. Urine Sampling and Biochemical Measurements

In order to assess the effect of water consumption on urinary acid-base parameters, participants were instructed to collect 24-hour urine samples on the day before each examination day and spontaneous urine samples on the examination days (*t*_0_, *t*_3_, and *t*_28_). All participants were given detailed written instructions for collecting the 24-hour urine samples and were provided with preservative-free plastic containers (Sarstedt AG and Co. KG, Nümbrecht, Germany). Participants were instructed to begin the collection the day before their scheduled examination, beginning after voiding and excluding the first morning urine. The collected urine included the morning sample of the following day. Subjects were asked to record both the start and end times of the collection on the plastic containers. To check the completeness of the 24-hour urine collection, urinary creatinine excretion was measured. Upon arrival at the research institute, urine volume was measured and urine samples were immediately mixed. They were then divided into aliquots, and stored at −22°C until analysis. One aliquot (1 urine tube) was stored at 5°C until transferred to an external laboratory (LADR, Laboratory Network Hanover) for further analysis. After the arrival at the research institute, subjects were asked to collect another urine sample, which served as spontaneous urine.

Urine pH was measured in both spontaneous urine and 24-hour urine samples using a pH meter (Mettler-Toledo, Giessen, Germany; Sartorius, Göttingen, Germany) at the Research Institute. Titratable acids (TA), ammonium (NH_4_^+^), and bicarbonate (HCO_3_^−^) were measured in 24-hour urine specimens according to the 3-step titration method by Lüthy [17]. These urine parameters were used to calculate renal net acid excretion (NAE) according to the following equation: NAE = TA − HCO_3_^−^ + NH_4_^+^.

### 2.6. Anthropometric Measurements

Anthropometric measurements were conducted at baseline (*t*_0_) and the end of the intervention period (*t*_28_). Height was measured with a stadiometer (Seca GmbH and Co. KG, Hamburg, Germany). Body weight was measured by the use of a digital scale (Kern and Sohn GmbH, Balingen-Frommern, Germany). BMI was calculated by dividing weight in kilograms by the square of height in meters. Waist and hip circumferences were measured at the end of a regular exhalation using a nonstretch tape.

### 2.7. Blood Sampling and Biochemical Measurements

To quantify biochemical indicators of acid-base status, venous blood samples were collected from each participant after a 12-hour overnight fast using EDTA, serum, and NaF citrate tubes (Sarstedt AG and Co. KG, Nümbrecht, Germany). Blood gas analysis was carried out at the research institute right after blood sampling. Blood pH, partial pressure of carbon dioxide (pCO_2_), base excess (BE), and bicarbonate (HCO_3_^−^) were analyzed using the epoc® Blood Analysis System (Siemens Healthcare GmbH, Erlangen, Germany). The remaining blood samples were stored at ≈5°C and transferred to an accredited and certified laboratory (LADR, Laboratory Network Hanover) for analysis.

### 2.8. Dietary Assessment and PRAL Calculation

To evaluate any potential dietary changes that may have occurred during the intervention, the participants' dietary intake was assessed before (*t*_0_) and at the end of the intervention period (*t*_28_) using 3-day food records. In order to directly compare nutritional intake and urinary output, participants recorded the type and amount of all foods and beverages consumed over three consecutive days (Friday, Saturday, and Sunday) directly before the first day of the examination (Monday). To take into account different mineralization of tap water, specific tap water composition of every participant was entered in the database. The composition of the participants' tap water was determined on the basis of the drinking water analysis of the water company that supplies the participants' particular area of residence. Nutrient intake was calculated from food and beverage intake (3 days). Moreover, the main fluid intake (beverages and soups) was evaluated at *t*_28_ to assess fluid balance. Nutrient and energy intake were estimated using the software PRODI 6.12 Expert® (Nutri-Science GmbH, Freiburg, Germany).

To evaluate the potential renal acid load (PRAL) of the nutrition, PRAL values of the food and beverages consumed were calculated using the formula proposed by Remer and Manz (PRAL_1995_) [18]. The PRAL value of the mineral water is calculated according to the formula adapted by Wynn [14].

Despite this, the following assumption was used to calculate the amount of salt ingested: NaCl = 40% Na and 60% Cl. Therefore, the calculation equation for salt was: Cl (mg) × 100/60 = NaCl (mg).

### 2.9. Statistical Analysis

Sample size calculation was made for the primary aim of the study (NAE) considering a statistical power of 80% and a hypothesized effect size above 1.0. An a priori sample size calculation revealed that 36 participants (18 for each water group) would be required to detect a statistical difference of *p* < 0.05 in a parallel group design.

Distribution of all data was assessed using Shapiro–Wilk test and Q-Q-plots. Non-normally distributed data were transformed using the natural logarithm (ln) or square root (sqrt) transformation, whichever was more appropriate. For data that exhibited non-normal distribution after transformation, nonparametric testing was used. Data are presented as mean ± standard deviation or median and interquartile range (IQR), depending on the distribution. The primary outcome measured is NAE. All other outcomes are secondary outcomes.

Differences in baseline characteristics between the two intervention groups were analyzed using the unpaired *t*-test or Mann–Whitney *U* test. Nominal variables were tested using Fischer's exact test.

In terms of nutritional data, between-group differences were analyzed using unpaired *t*-test or Mann–Whitney *U* test, while within-group differences were analyzed using paired *t*-test or Wilcoxon test. Moreover, a two-way repeated measures ANOVA was computed to detect intervention effects (interactions between time and group), despite the partial absence of normal distribution and the partial presence of heteroscedasticity. According to Bortz [19], variance analyses are considered robust against violation of the normal distribution assumption with a sufficiently large sample size (central limit theorem, *n* > 50), and inhomogeneous variances are not a problem with approximately equal group sizes. In addition, repeated-measures analyses of variance (rmANOVA) followed by Bonferroni correction for multiple testing (pairwise testing) were also performed between baseline (*t*_0_), interim (*t*_3_), and final (*t*_28_) examinations to compare inner-group differences. Two-sided *p* values <0.05 were considered as statistically significant. All statistical analyses were performed using SPSS Software for Windows (Version 28.0.1.0; SPSS Inc., Chicago, IL, USA).

## 3. Results

### 3.1. Study Population

**Figure 1:**
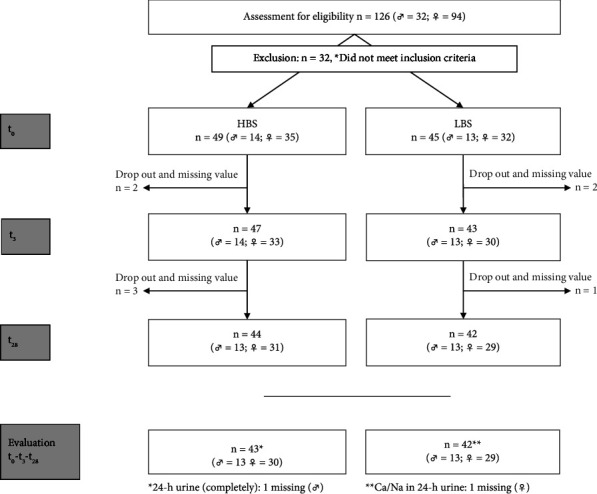
Flowchart of the study.

The allocation of participants to the two intervention groups is shown in Figure 1. Ninety-four participants (67 women, 27 men) were included in this study, but 9 subjects were excluded from the evaluation due to dropouts and missing values (forgotten samples, illness or other personal reasons). One subject in the HBS group withdrew from the study due to gastrointestinal discomfort. No participant was excluded due to insufficient water consumption. Therefore, data from 85 participants (HBS group: *n* = 43, LBS group: *n* = 42) were analyzed. In terms of 24-hour urine data, there was one more missing data in the HBS group, resulting in 42 evaluable 24-hour urine samples.

General baseline characteristics of both intervention groups are presented in Table 2. At baseline, 59 women (69.4%) and 26 men (30.6%) aged 53 ± 10 years were included in the study. There were no significant differences in anthropometric parameters between the two intervention groups, and these parameters did not change significantly from the beginning to the end of the intervention. Participants maintained their physical activity levels during the intervention period.

### 3.2. Dietary Intake of PRAL-Related Nutrients and Dietary Acid Load

Intake of macronutrients as well as PRAL-related micronutrients is summarized in Table 3. In both intervention groups, the intake of macronutrients did not change significantly between baseline (*t*_0_) and final examination (*t*_28_), with no significant interaction between time and group. Moreover, there were no significant changes in micronutrient intake with respect to calcium, magnesium, and phosphorus intake in both intervention groups, with no significant interaction between time and group. On the contrary, the intake of sodium, chloride, and potassium differed between baseline (*t*_0_) and final examination (*t*_28_), depending on the intervention group. In the HBS group, sodium and chloride intake was significantly higher at the end of the study (*t*_28_) compared to baseline (*t*_0_), as a result of test water consumption (*p* < 0.001 and *p*=0.001, respectively). In the LBS group, there were no significant changes in sodium intake, but chloride intake was significantly lower at the end of the intervention (*t*_28_) (*p*=0.025). Consequently, there was a significant change in salt consumption (NaCl) in both groups, but showing opposite directions. While the consumption of NaCl in the HBS group was significantly higher at the end of the intervention (*t*_28_) compared to baseline (*t*_0_) (*p*=0.001), it was significantly lower in the LBS group at the end of the intervention (*t*_28_) (*p*=0.025). Furthermore, potassium intake in the LBS group was significantly lower at the end of the intervention (*t*_28_) compared to baseline (*t*_0_) (*p*=0.002).

Moreover, PRAL values calculated from dietary protocols (PRAL_1995_) showed a significant reduction of dietary acid load in the HBS water group (*p* < 0.001) and no change in the LBS water group (*p*=0.200).

### 3.3. Bicarbonate and Salt Intake from Mineral Water

The two different mineral waters provided different amounts of minerals. While the HBS group consumed a high amount of additional bicarbonate, sodium, and chloride from the test water, the additional amounts of minerals from the consumed water in the LBS group were very low. While participants in the HBS group consumed an additional 6552 mg/d of bicarbonate, participants in the LBS group consumed an additional 342 mg/d of bicarbonate from the study water (1,500 mL). The consumption of 1,500 mL HBS water led to an additional intake of 2.56 grams of sodium and 0.48 grams of chloride per day, while the consumption of LBS water led to an additional intake of 0.013 grams of sodium and 0.017 grams of chloride per day. Despite the different amounts of sodium and chloride, blood pressure did not change differently between the two water groups (data published elsewhere) [20]. With the exception of potassium and sulfate, which were present in higher concentrations in HBS mineral water (Table 1), no other constituent of the two mineral waters led to substantial different ingested amounts in minerals.

### 3.4. Effect of Mineral Water on Urine Volume and Urinary Acid-Base Parameters

Urine volume did not change differently between the water groups (*p*=0.176). Both groups showed an increase in urine volume of about 400 mL within the first 3 days of water consumption (*p*=0.002 and *p* < 0.001 for HBS and LBS group, respectively) followed by stable urine volume until the end of the intervention (*t*_28_) (*p*=1.000 and *p*=0.222 for HBS and LBS group, respectively). Consequently, long-term assessments also showed an increase in urine volume (*p* < 0.001 and *p* < 0.012 for HBS and LBS group, respectively). There was no significant difference in urine volume between groups at any time point (data not shown).

Daily mineral water consumption in the HBS group was 1,625 (214) mL of HBS mineral water, while LBS mineral water consumption in the LBS group was 1,643 (286) mL. At the end of the intervention, fluid intake and urinary excretion was balanced in both groups. Participants in the HBS group consumed 2,704 (1,100) mL of main fluids (beverages + soups), while the LBS group consumed 2,605 (822) mL. As shown in Table 4, urinary output was 2,687 (1,081) mL and 2,704 (1,021) mL, respectively. A comparison of fluid intake and urine volume showed no significant differences within both groups (*p*_HBS_=0.671, *p*_LBS_=0.512).

As shown in Table 4, the effect of mineral water consumption on urinary acid-base parameters was substantial, with significant effects were primarily evident in the HBS group.

All urinary acid-base parameters showed significant interactions between time and group (all *p* < 0.001), indicating different effects of water consumption in the two intervention groups. While the consumption of HBS water resulted in significant changes in all urinary acid-base parameters (all *p* < 0.001), the consumption of LBS water significantly affected only a few parameters.

**Figure 2:**
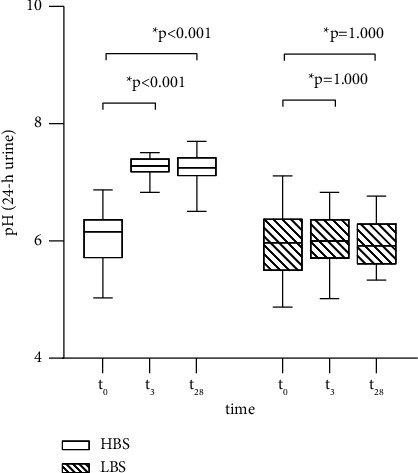
pH of 24-hour urine at the beginning of the study (*t*_0_), at the interim examination (*t*_3_), and at the end of the intervention (*t*_28_).

Specifically, in the HBS group, urinary pH (24-hour urine and spontaneous urine) and HCO_3_^−^ increased significantly within the first 3 days of water consumption (all *p* < 0.001), followed by a stable high level until the end of the intervention (*t*_28_) (*p*_24−h urin_=1.000, *p*_spot urine_=0.097, *p*_HCO3−_=1.000). Consequently, long-term consumption of HBS water also resulted in significant increases in urine pH (24-hour urine and spontaneous urine) and HCO_3_^−^ (all *p* < 0.001) of approximately the same magnitude as in the short-term period (Figure 2). In contrast, in the LBS group, HCO_3_^−^ decreased slightly within the first 3 days of water consumption (*p*=0.009), followed by a stable period until the end of the intervention (*t*_28_) (*p*=0.805). However, long-term consumption of LBS water showed no significant effect (*p*=0.092).

Moreover, TA and NH_4_^+^ decreased significantly within the first 3 days of water consumption in both intervention groups (HBS: both *p* < 0.001; LBS: *p*_TA_=*p*=0.028, *p*_NH4+_ < 0.001), followed by almost stable levels until the end of the intervention (*t*_28_) (HBS: *p*_TA_=1.000, *p*_NH4+_=0.131; LBS: *p*_TA_=0.129, *p*_NH4+_=0.830). However, the decrease in both TA and NH_4_^+^ was significantly greater in the HBS group compared to the LBS group. In the HBS group, TA levels were almost negligible, while NH_4_^+^ was reduced by half. Again, long-term consumption of HBS water resulted in significant reductions of TA and NH_4_^+^ (both *p* < 0.001) of approximately the same magnitude as in the short-term period. In contrast, only NH_4_^+^ was significantly reduced in the LBS group after long-term consumption of mineral water (*p*=0.023). However, these declines were smaller than those in the HBS group. In addition, TA in the LBS group showed no significant changes over the long-term period of consumption (*p*=0.520).

**Figure 3:**
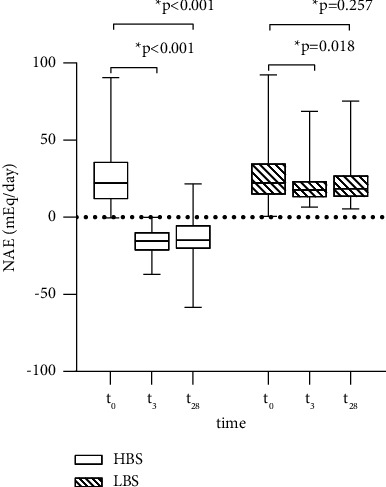
NAE at the beginning of the study (*t*_0_), at the interim examination (*t*_3_), and at the end of the intervention (*t*_28_).

Finally, this resulted in a significant interaction between time and group for NAE (*p* < 0.001), indicating different effects of water consumption in the two water groups (Figure 3). Nevertheless, in both intervention groups, NAE significantly decreased within the first 3 days of water consumption (*p* < 0.001 and *p*=0.018 for HBS and LBS group, respectively). However, the reductions were more pronounced in the HBS group than in the LBS group. From the interim examination (*t*_3_) until the end of the intervention (*t*_28_), both groups showed a stable NAE (*p*=0.875 and *p*=0.377 for HBS and LBS group, respectively). Moreover, long-term consumption of both mineral waters showed a decline in NAE levels, which were only significant in the HBS group (*p* < 0.001 and *p*=0.257 for HBS and LBS group, respectively).

### 3.5. Effect of Mineral Water on Blood Parameters of Acid-Base-Status

As shown in Table 5, the effect of mineral water consumption on venous blood gas parameters was modest, with significant effects observed primarily in the HBS group.

In blood samples, pH and pCO_2_ did not show a significant interaction between time and group (*p*=0.145 and *p*=0.303, respectively), indicating similar effects of water consumption in the two intervention groups. However, pH in the HBS group tended to increase during the initial 3 days of water consumption (*p*=0.068) and significantly decreased from the interim examination (*t*_3_) to the end of the intervention (*t*_28_) (*p* < 0.001); no long-term changes were observed (*p*=0.427). In the LBS group, only long-term changes were significant (*p*=0.036), showing a decrease in pH. For pCO_2_, the HBS group showed a slight but nonsignificant increase during the first 3 days of water consumption (*p*=0.807) and from the interim (*t*_3_) examination to the end of the intervention (*t*_28_) (*p*=0.167), with a significant increase during the long-term consumption (*p*=0.027). In the LBS group there were no significant changes.

Moreover, for HCO_3_^−^ and BE significant time and group interactions were shown (*p*=0.001 and *p* < 0.001, respectively), indicating different effects of water consumption. Differences were significant only in the HBS group (both *p* < 0.001). Specifically, HCO_3_^−^ and BE levels significantly increased during the initial 3 days of water consumption (*p*=0.004), followed by a slight, but nonsignificant decrease of HCO_3_^−^ (*p*=1.000) and a significant decrease of BE (*p*=0.015) until the end of the intervention (*t*_28_). Long-term consumption of the HBS water also led to a significant increase in HCO_3_^−^ and BE levels (*p*=0.011 and *p*=0.030, respectively).

## 4. Discussion

In this study, the daily consumption of 1500–2000 mL of bicarbonate- and sodium-rich mineral water led to significant positive changes in urinary and blood acid-base parameters. On the contrary, drinking the LBS water showed only slight effects. The favorable outcomes of consuming high-bicarbonate-sodium (HBS) water were observed after a short-term consumption period (3 days) and maintained until the end of the study (28 days). Throughout the follow-up period, the majority of parameters in both blood and urine remained stable, maintaining similar levels to those observed after the initial short-term intervention.

Considering that the diet remained unchanged, except for the main beverages, it is reasonable to conclude that the observed differences are not due to alterations in dietary intake. Instead, these differences can be exclusively attributed to the impact of the mineral water.

The long-term results of the present study are consistent with previous mineral water studies showing positive effects on acid-base balance after consumption of bicarbonate-rich mineral water [10, 13, 14, 21–26]. Except one study, which evaluated effects on several acid-base parameters in blood and urine samples [10], most of the conducted mineral water studies concentrated on blood and/or urinary pH. Blood gas parameters and net acid excretion (NAE) are not commonly assessed in studies involving mineral water, with only three exceptions [10, 13, 25] having delved into this area. Nonetheless, several studies have highlighted the favorable impacts of HCO_3_^−^ supplementation on blood HCO_3_^−^ levels [27–31], blood BE [30], and urinary NAE [32, 33]. Moreover, short-term HCO_3_^−^ supplementation studies have demonstrated positive effects on urinary pH [34, 35].

### 4.1. Effects on Urine Volume and Urinary Acid-Base Parameters

In the current study, urine volume increased significantly in both intervention groups. At the end of the study, both groups showed a neutral fluid balance, with no significant differences between the main fluid intake and urinary fluid excretion. However, considering that the body eliminates water not solely through urine but also via skin, feces, and respiration [36], a slightly negative fluid balance could be assumed according to the presented data. Nevertheless, fluids may have been balanced, because measured fluid intake just covered beverages and fluid intake from soups. Additional fluid intake from fruits, legumes, and other foods was not evaluated, leading to a lower reported fluid intake than actually consumed. Moreover, there were no differences in urine volume between the two study groups at all time points. This indicated that both study groups underwent comparable dilution effects due to urine volume. The findings align with previous mineral water studies, where significant increases have been observed in response to specific daily intakes ranging from 1,500 ml to 2,000 ml of mineral water [10, 14, 22–24]. With the exception of one study, the magnitudes of increase did not diverge between intervention groups within each study. In the mentioned study, the group consuming bicarbonate-rich mineral water displayed an elevation in urine volume during the initial 4 weeks, followed by a reduction in the magnitude of increase almost returning to baseline levels. Conversely, the control groups exhibited minor declines in volume during the initial 8 weeks, followed by a slight increase by the end of the intervention [23]. In addition, only one study showed a reduction in urine volume after the consumption of alkalizing mineral water [13].

Furthermore, impacts on urinary acid-base parameters include changes in pH (24-hour urine and spontaneous urine), TA, HCO_3_^−^, and NH_4_^+^. The latter three parameters can be summarized as urinary NAE, serving as an indicator for dietary acid load [37]. As mentioned above, urinary parameters were strongly influenced by the consumption of HBS water within this study. The very high HCO_3_^−^ intake through HBS water resulted in an excess of HCO_3_^−^ being excreted via urine. The buffering effect was substantial; TA became virtually undetectable. All effects were highly significant in the short-term and in the long-term analysis. For pH in 24-hour urine, these findings are consistent with the results of several previous mineral water studies [10, 13, 14, 21–26]. In the previous studies, pH in 24-hour urine increased by about 0.29 to 0.80 points (mean), whereas the consumption of HBS water resulted in an even more pronounced pH increase of about 1.19 points (mean). Concerning spontaneous urine, only a single prior mineral water study has examined the effect on pH, demonstrating comparatively smaller positive effects than those seen after HBS water consumption [10]. Regarding acid-base parameters determined by titration, there are only a few previous studies evaluating the impact on these parameters in mineral water studies [10, 14, 22], all showing similar effects. In the largest study conducted with 129 healthy participants, three different bicarbonate-rich mineral waters with different PRAL values (MBMP water, HBLP water, and MBLP water) were tested against low-bicarbonate mineral water with a high PRAL value (LBHP water) [10]. While MBMP water was characterized by a moderate HCO_3_^−^ content and a medium PRAL value, HBLP water had a high HCO_3_^−^ content and a low PRAL value, and MBPL water had a medium HCO_3_^−^ content and a low PRAL value. In this study, the consumption of the bicarbonate-rich mineral waters with different PRAL values resulted in favorable effects on urinary TA, HCO_3_^−^, NH_4_^+^, and NAE compared to the low-bicarbonate mineral water with a high PRAL value. Nonetheless, some distinctions emerged among the three bicarbonate-rich mineral waters used in this study. The positive changes in urinary parameters were more pronounced in the groups drinking bicarbonate-rich mineral water with a low PRAL value (HBLP and MBLP water). The researchers concluded that alkalinity (PRAL value in mineral water) has a greater influence on acid-base parameters than HCO_3_^−^ content [10]. Under the same presumption, it was anticipated that the acid-base balance in the current study would be more profoundly affected by HBS water consumption in the current study. This was attributed to its higher alkalinity (PRAL −63.07), resulting from a high sodium content. In fact, although the effects on urinary parameters showed the same direction in both studies, the effects of HBS water consumption surpassed those exhibited by the waters investigated by Wasserfurth et al. [10].

It is noteworthy that baseline NAE was lower than expected. Typically, individuals following an omnivorous Western diet display a NAE ranging between 70 and 100 mEq/day [38]. In contrast, in both intervention groups, NAE was about 27 mEq/day at baseline. There are two explanations for such a low NAE. Firstly, the study population may have been more health conscious than the general population. They may have consumed a more healthy and therefore less acidic diet than the average population. Lower NAE values have already been reported with a plant-based diet [39]. Moreover, there are several studies that have reported similar baseline NAE values as reported in the current study following omnivorous diets [10, 40–42]. Secondly, urinary bicarbonate, a component of NAE calculations, may have been somewhat overestimated by titration of HCO_3_^−^ [17]. Considering the initial low NAE levels and the substantial buffering impact on urinary acid-base parameters, the substantial reduction in NAE to negative values is not surprising. The negative NAE is consistent with a previous study showing that certain diets can lead to negative values [37].

Despite the direct influence of diet on NAE, several hormones are involved in the regulation of renal acid-base excretion. Higher levels of aldosterone, angiotensin II, endothelin, parathyroid hormone, and glucocorticoids are linked to higher renal acid excretion. Moreover, metabolic acidosis has been shown to increase hormonal activity [43]. Therefore, the correction of the slightly low-grade metabolic acidosis may have involved a change in hormonal regulation leading to a change in TA, HCO_3_^−^, and NH_4_^+^excretion, possibly caused by the mineral water consumption. Nevertheless, this is speculative, as only aldosterone has been examined in the present study. As previously reported, the consumption of bicarbonate- and sodium-rich mineral water in this study resulted in a significant decrease in urinary aldosterone [20], which may have contributed to a lower NAE.

### 4.2. Effects on Venous Acid-Base Blood Gas Parameters

Effects on acid-base blood gas parameters include pH, BE, HCO_3_^−^, and pCO_2_. This is the first study showing beneficial effects of short-term and long-term consumption of mineral water very high in bicarbonate and high in sodium on venous blood gas parameters.

As mentioned above, long-term effects have already been demonstrated in previous studies [10, 13, 25] and could be supported by this study results. In line with our findings, the study carried out by Wasserfurth et al. [10] similarly indicated no noteworthy shifts in venous blood pH after a 4-week consumption of three different bicarbonate-rich mineral waters possessing different PRAL values. Nevertheless, in the study presented here, pH values in blood tended to increase over the first three intervention days. This may also have occurred in the Wasserfurth study, but it could not be determined due to the long-term design of the study. Moreover, the short-term results of the current study are similar to the results of a previous intervention study with an alkaline mineral water (pH 10). In that study, the consumption of mineral water resulted in a significant increase in blood pH after a rather short duration of 2 weeks [13]. However, capillary blood was used. Although arterial blood analyses are required for the evaluation of respiratory conditions, metabolic disturbances can also be adequately measured in venous blood samples [44, 45]. Venous pH, bicarbonate, and base excess closely align with arterial values. However, the agreement between arterial and venous pCO_2_ is inconsistent [44]. Under normal physiological conditions, blood pH is kept in a narrow range between 7.35 and 7.45 (arterial pH) to maintain physiological processes in the body, mediated by intracellular and interstitial pH (4.8). The varying effects of bicarbonate-rich mineral water on blood pH in the short term and long term may be the result of efficient buffering systems to maintain a stable blood pH. Although drinking mineral water can lead to a transient pH increase, buffering systems react sequentially to keep it within the normal range over the long term.

In addition, within the scope of this study, there was a noteworthy increase in HCO_3_^−^ and BE during the initial 3-day phase of mineral water consumption and these elevated concentrations remained until the end of the intervention. This illustrates that the impact of consuming mineral water on blood acid-base balance becomes apparent shortly after the first consumption and continues to endure over a long period of time, without any habituation effects. These findings align with the outcomes of the study carried out by Wasserfurth et al. [10]. In that research, noteworthy impacts on HCO_3_^−^ and BE were exclusively observed in the subset consuming mineral water with a moderately high HCO_3_^−^ content and a low PRAL value (MBLP water, PRAL = −22.1). However, the group consuming bicarbonate-rich mineral water with a low PRAL (HBLP water, PRAL = −19.3) also displayed a tendency towards an increase in these parameters. Notably, no distinctions were evident for the group consuming bicarbonate-rich mineral water with a moderate PRAL value (MBMP water, PRAL = −10.8). The authors conclude that alkalinity may be more important than bicarbonate in regulating acid-base balance in blood. Based on this assumption and considering that the PRAL value of the water consumed in this current study (HBS water, PRAL = −63.07) was even lower than the PRAL value of the HBLP water in Wasserfurth's study, a greater impact on blood gas parameters was anticipated in the present study. Nevertheless, the effect on HCO_3_^−^ and BE was slightly lower with HBS water than the effect of low-PRAL mineral water in Wasserfurth's study (MBLP and HBLP group).

Although pCO_2_ significantly increased from the beginning of the intervention to the end, changes were marginal following HBS water consumption. In contrast, the changes on pCO_2_ in the MBHP group were more pronounced in the mineral water study by Wasserfurth et al. [10].

### 4.3. Effects on Blood Pressure

Both mineral waters contained different amounts of sodium. The evaluation of the changes in blood pressure due to mineral water consumption has been published elsewhere [20]. For the sake of completeness, the blood pressure results are also mentioned here. Despite the different amounts of sodium, blood pressure did not change differently between the two water groups. Furthermore, no differences were found between men and women, or between younger (<50 years) and older (≥50 years) participants in either intervention group. This is in line with the results of other studies in which the consumption of bicarbonate- and sodium-rich mineral water did not lead to blood pressure changes [26, 46–48].

### 4.4. Implications for Human Health

Urine pH of healthy subjects ranges usually between 5.8 and 6.8, depending on the consumed diet [49]. It has been shown that vegans have a higher urine pH (6.7) than omnivores (6.2) [50]. This is in line with our findings in the LBS group, where urine pH was about 6 and did not change significantly over the intervention period. Thus, mineral water consumption did not modify acid-base balance in this group. Moreover, NAE was only slightly positive in this group, indicating a moderate acidic diet. Since included subjects were without impaired kidney function, it is reasonable to assume that the excess acid from the diet would have been excreted without negative effects on human health. However, a lower urine pH value, associated with a higher NAE, can promote the formation of certain kidney stones [51]. In addition, higher NAE values are associated with inflammatory processes in the kidneys [52].

Base supplements and bicarbonate-rich mineral waters are commonly used for the modulation of urine composition. Their alkalizing effect has been proven in several studies until now [10, 14, 22]. Therefore, the most obvious impact on human health relates to the modulation of kidney stone risk (calcium oxalate stones, uric acid stones) due to changes in urinary pH. While a low urine pH favors crystallization, an elevated urine pH counteracts crystallization [49, 53]. Moreover, bicarbonate-rich mineral water has been shown to improve urinary inhibitors for crystallization, mainly citrate and magnesium [49]. Accordingly, the consumption of bicarbonate-rich mineral water has a positive effect on the risk of calcium oxalate stones and uric acid stones due to its urine alkalizing effect and changes in urine composition.

In addition, as demonstrated by the lowering of NAE, bicarbonate-rich mineral water has a positive impact on acid-base-balance [10]. Therefore, the negative effects of an acidic diet can be mitigated by its intake. On the one hand, a high intake of acid-producing precursors has been shown to negatively affect bones [54]. On the other hand, the intake of bicarbonate-rich mineral water has been shown to lower bone resorption [14, 25, 55]. Thus, it could be assumed that the consumption of bicarbonate-rich mineral water has a positive effect on bones due to its improvement in acid-base balance.

### 4.5. Strengths and Limitations

Within the context of this study, there are some strengths and limitations that should be mentioned. One limitation is the absence of blinding in the study design which may have an influence on the study outcome. Since the taste of the water alone indicated the group assignment, blinding was not feasible. Moreover, the lack of meal standardization should be recognized as a possible source of influence on the study outcomes. The inclusion of such standardized meals would have been beneficial to increase the robustness of our results. This approach would have taken into account the different effects of different meals on urinary acid excretion, thus providing more reliable results. Furthermore, it would have been beneficial to include additional control groups consuming mineral water high in sodium alone. This would have allowed for a more precise evaluation of the individual effects of HCO_3_^−^ and PRAL of the mineral water and improved the study's ability to make specific statements regarding their respective impacts.

Conversely, it is important to acknowledge the strengths of this study. A strength is the sample size, surpassing previous studies that examined similar effects with smaller participant groups. This increased sample size enhances the study's statistical power, making it more likely to detect true effects and enabling more precise estimates of the observed effects. Furthermore, assessing effects at multiple time points provides the opportunity to capture progression over time and uncover potential habituation effects. Another advantage of the study is the extensive data collection on various parameters, including blood gas and urinary parameters, providing a comprehensive view of potential effects.

## 5. Conclusion

In conclusion, this study highlights the significant and beneficial effects of mineral water consumption, characterized by a very high bicarbonate and high sodium content (HBS water) content, on acid-base parameters. In particular, the consumption of 1,500 to 2,000 mL mineral water led to a significant reduction in urinary NAE, as reflected by decreased levels of TA and NH_4_^+^, with a concomitant increase in urinary HCO_3_^−^ excretion. A significant increase in urinary pH was also observed. Moreover, blood gas parameters were positively affected, although to a lesser extent. Taken together, these results emphasize that the consumption of mineral water with high HCO_3_^−^ and sodium concentrations exerts a beneficial effect on the acid-base balance in humans. These effects became apparent shortly after the beginning of consumption and showed no signs of habituation. Moreover, no adverse effects on human health have been observed.

Further research should address the different effects on acid-base parameters of consuming mineral water with a very low PRAL and simultaneous low bicarbonate content compared to a bicarbonate-rich mineral water. This research is warranted to isolate the potential effects of the co-occurrence of high HCO_3_^−^ content and low PRAL.

## Figures and Tables

**Table 1:** Mineral content and PRAL of the test products.

Mineral	Test product	
	HBS mineral water	LBS mineral water
HCO_3_^−^ (mg/L)	4368	228
Na^+^ (mg/L)	1708	8.4
Cl^−^ (mg/L)	322	11
SO_4_^2−^ (mg/L)	174	15
K^+^ (mg/L)	110	2.3
Ca^2+^ (mg/L)	90	67.5
Mg^2+^ (mg/L)	11	6.9
P (mg/L)	0.0^*∗∗*^	0.0^*∗∗*^
PRAL (mEq/L)	−63.07	−0.89

HBS = high bicarbonate and sodium, LBS = low bicarbonate and sodium. PRAL = potential renal acid load according to the formula proposed by Wynn et al. [14]. ^*∗∗*^Phosphate content below detection limit.

**Table 2:** Baseline (*t*_0_) characteristics of the study groups.

Parameter	HBS group *n* = 43	LBS group *n* = 42	*p* value^*∗*^
Women/Men	30/13	29/13	0.943
Age (years)	55 (17)	55 (10)	0.725
Body weight (kg)	70.5 (19.3)	69.8 (17.9)	0.582
Height (m)	1.70 (0.12)	1.70 (0.10)	0.287
BMI (kg/m^2^)	23.8 (4.51)	24.6 (5.63)	0.529
WC (cm)	85.0 (14.0)	83.5 (18.0)	0.519
HC (cm)	99.0 (12.0)	101 (9.00)	0.748
WHR	0.83 (0.09)	0.85 (0.11)	0.547

Data are shown as median (IQR). HBS = high bicarbonate and sodium, LBS = low bicarbonate and sodium. BMI = body mass index, HC = hip circumference, WC = waist circumference, WHR = waist-to-hip ratio. ^*∗*^Distribution of gender between groups was analyzed using the Chi-Quadrat test. All other group differences were assessed using unpaired *t*-test/Welch-test, or Mann–Whitney *U* test.

**Table 3:** Dietary energy and nutrient intake calculated from 3-day dietary records at the beginning (*t*_0_) and at the end of the intervention (*t*_28_) as well as absolute changes (∆).

Parameter	Group	Time		Change	*p* value (time × group)^*∗*^	*p* value (time)^*∗*^
		*t*_0_	*t*_28_	∆ 28-0		
Fiber (g/d)	HBS	23.4 (14.7)	25.5 (10.2)	1.53 (7.73)	0.441	0.452
	LBS	19.8 (13.4)	20.9 (11.1)	−0.85 (8.97)		0.593

Protein (g/d)	HBS	83.0 (29.3)	80.4 (24.5)	−1.76 (25.9)	0.330	0.832
	LBS	77.5 (32.4)	70.9 (28.0)	−4.71 (29.2)		0.109

Fat (g/d)	HBS	99.0 (53.8)	101 (30.0)	−1.85 (42.6)	0.284	0.928
	LBS	89.4 (35.6)	82.8 (49.6)	−4.27 (39.9)		0.160

Carbohydrates (g/d)	HBS	234 (91.9)	228 (77.3)	15.0 (75.2)	0.922	0.646
	LBS	266.4 (79.9)	202.9 (87.2)	−2.76 (78.1)		0.365

Energy (kJ/d)	HBS	9687 (3093)	9525 (2454)	−249 (2648)	0.414	0.552
	LBS	9436 (2957)	8629 (3319)	−550 (3234)		0.120

Calcium (mg/d)	HBS	932 (515)	1002 (423)	−24.2 (352)	0.820	0.938
	LBS	862 (396)	860 (488)	49.0 (393)		0.861

Chloride (mg/d)	HBS	4240 (1556)	4952 (1720)	709 (1472)	**<0.001**	**0.001**
	LBS	4128 (2097)	3240 (1568)	−386 (1706)		**0.025**

Potassium (mg/d)	HBS	3286 (771)	3444 (930)	214 (1064)	**0.002**	0.303
	LBS	3411 (1650)	2713 (1183)	−582 (1070)		**0.002**

Magnesium (mg/d)	HBS	378 (164)	383 (135)	−11.6 (133)	0.581	0.626
	LBS	378 (171)	343 (155)	−32.4 (158)		0.218

Sodium (mg/d)	HBS	2706 (884)	5880 (1207)	3078 (1073)	**<0.001**	**<0.001**
	LBS	2601 (1155)	2249 (958)	−146 (1457)		0.128

Phosphorus (mg/d)	HBS	1429 (631)	1377 (457)	−43.0 (415)	0.838	0.809
	LBS	1304 (482)	1197 (493)	−26.5 (517)		0.507

NaCl (mg/d)	HBS	7066 (2593)	8254 (2867)	1182 (2453)	**<0.001**	**0.001**
	LBS	6880 (3495)	5400 (2613)	−643 (2844)		**0.025**

PRAL_1995_ (mEq/d)	HBS	3.89 (26.35)	−107 (35.2)	−110 (29.1)	**<0.001**	**<0.001**
	LBS	−0.89 (19.14)	1.23 (14.32)	2.82 (22.18)		0.200

Data are shown as median (IQR). HBS = high bicarbonate and sodium, LBS = low bicarbonate and sodium. PRAL = potential renal acid load. ^*∗*^Time × group interactions were analyzed using two-way repeated measures ANOVA. ^*∗∗*^Differences between *t*_0_ and *t*_28_ within each group were analyzed using paired *t*-Test and Wilcoxon-Test. The bold values are significant at p <0.05.

**Table 4:** Urine analysis and fluid consumption (main fluids) at the beginning of the study (*t*_0_), at the interim examination (*t*_3_), and at the end of the intervention (*t*_28_) as well as absolute changes (∆).

Parameter	Group	Time			*p* value (time × group)^*∗*^	*p* value (time)^*∗*^	Change	
		*t*_0_	*t*_3_	*t*_28_			∆ 3-0	∆ 28-0
Fluid consumption (mL)	HBS	2300 (1065)^c^	—	2704 (1100)^c^	0.255	**<0.001**	—	520 (810)
	LBS	2260 (920)^c^	—	2605 (822)^c^		**0.010**	—	175 (1175)

Urine volume (mL)	HBS	2341 (1221)^a,c^	2742 (879)^a^	2687 (1081)^c^	0.176	**<0.001**	395 (815)	365 (962)
	LBS	2193 (1098)^a,c^	2685 (951)^a^	2704 (1021)^c^		**<0.001**	402 (943)	363 (1214)

pH (24-hour urine)	HBS	6.17 (0.66)^a,c^	7.28 (0.22)^a^	7.23 (0.32)^c^	**<0.001** ^†^	**<0.001** ^†^	1.15 (0.77)	1.15 (0.65)
	LBS	5.98 (0.88)	6.00 (0.66)	5.92 (0.68)		0.710	0.07 (0.96)	−0.02 (0.53)

pH (spontaneous urine)	HBS	5.66 (0.58)^a,c^	7.07 (0.53)^a^	6.90 (0.71)^c^	**<0.001**	**<0.001**	1.36 (0.88)	0.97 (0.78)
	LBS	5.68 (0.99)	5.65 (0.86)	5.53 (0.91)		0.922	−0.05 (0.74)	−0.04 (0.92)

TA (mmol/L)	HBS	7.78 (13.85)^a,c^	0.00 (0.00)^a^	0.00 (0.00)^c^	**<0.001** ^†^	**<0.001** ^†^	−7.78 (13.85)	−7.78 (13.10)
	LBS	9.20 (11.75)^a^	6.95 (5.35)^a^	8.33 (6.75)		0.152	−2.43 (10.35)	−0.68 (10.70)

HCO_3_^−^ (mmol/L)	HBS	8.25 (4.00)^a,c^	24.73 (9.00)^a^	24.50 (12.53)^c^	**<0.001** ^†^	**<0.001**	16.48 (10.33)	13.68 (16.90)
	LBS	6.83 (6.15)^a^	6.18 (2.65)^a^	6.18 (2.25)		**0.004**	−1.43 (2.50)	−0.74 (3.37)

NH_4_^+^ (mmol/L)	HBS	22.15 (12.85)^a,c^	10.70 (5.00)^a^	11.50 (5.60)^c^	**<0.001**	**<0.001**	−9.68 (12.95)	−9.81 (12.73)
	LBS	20.45 (11.48)^a,c^	17.25 (6.70)^a^	17.05 (8.57)^c^		**<0.001**	−3.94 (8.55)	−2.68 (11.80)

NAE (mEq/d)	HBS	22.44 (23.80)^a,c^	−15.40 (11.40)^a^	−14.25 (15.00)^c^	**<0.001** ^†^	**<0.001** ^†^	−37.29 (23.25)	−36.72 (20.57)
	LBS	22.28 (19.00)^a^	17.80 (9.65)^a^	18.53 (13.20)		**0.011** ^†^	−5.68 (17.00)	−3.81 (18.15)

Data are shown as median (IQR). HBS = high bicarbonate and sodium, LBS = low bicarbonate and sodium. TA = titratable acids, HCO_3_^−^ = bicarbonate in urine, NH_4_^+^ = ammonium, NAE = net acid excretion. ^*∗*^Time × group interactions were analyzed using two-way repeated measures ANOVA. ^*∗∗*^Differences over the intervention time within each group were assessed using repeated measures ANOVA. ^†^Greenhouse–Geisser or Huynh–Feldt adjustment was used to correct for violations of sphericity. ^a^ = sign. Differences between *t*_0_ and *t*_3_ (short term), ^b^ = sign. Differences between *t*_3_ and *t*_28_ (follow-up), ^c^ = sign. Differences between *t*_0_ and *t*_28_ (long-term). The bold values are significant at p <0.05.

**Table 5:** Blood gas analysis at the beginning of the study (*t*_0_), at the interim examination (*t*_3_), and at the end of the intervention (*t*_28_) as well as absolute changes (∆).

Parameter	Group	Time			*p* value (time × group)^*∗*^	*p* value (time)^*∗*^	Change	
		*t*_0_	*t*_3_	*t*_28_			∆ 3-0	∆ 28-0
pH	HBS	7.381 (0.035)	7.396 (0.048)^b^	7.382 (0.045)^b^	0.145	**<0.001**	0.01 (0.04)	−0.01 (0.03)
	LBS	7.397 (0.036)^c^	7.383 (0.039)	7.374 (0.037)^c^		**0.027**	0.00 (0.04)	0.00 (0.04)

HCO_3_^−^ (mmol/L)	HBS	25.6 (2.70)^a,c^	26.00 (2.60)^a^	25.80 (3.20)^c^	**0.001**	**<0.001**	0.60 (2.50)	0.90 (2.20)
	LBS	25.6 (2.50)	25.00 (2.10)	25.10 (1.50)		0.428	−0.30 (1.70)	−0.40 (1.80)

BE (b) (mmHg)	HBS	0.20 (2.30)^a,c^	0.90 (2.60)^a,b^	0.80 (2.00)^b,c^	**<0.001**	**<0.001**	0.80 (1.50)	0.70 (1.70)
	LBS	0.20 (1.40)	−0.15 (2.00)	−0.05 (1.30)		0.146	−0.15 (1.30)	−0.45 (1.80)

pCO_2_ (mmHg)	HBS	43.40 (7.70)^c^	43.40 (8.00)	43.60 (9.10)^c^	0.303	**0.016**	1.20 (6.80)	1.90 (5.40)
	LBS	41.85 (7.40)	41.10 (5.50)	43.00 (5.90)		0.132	−0.70 (6.40)	−0.15 (5.10)

Data are shown as median (IQR). HBS = high bicarbonate and sodium, LBS = low bicarbonate and sodium. BE = base excess, HCO_3_^−^ = bicarbonate in venous blood, pCO_2_ = partial pressure of carbon dioxide in venous blood. ^*∗*^Time × group interactions were analyzed using two-way repeated measures ANOVA. ^*∗∗*^Differences over the intervention time within each group were assessed with repeated measurements ANOVA. ^a^ = sign. Differences between *t*_0_ and *t*_3_ (short term), ^b^ = sign. Differences between *t*_3_ and *t*_28_ (follow-up), ^c^ = sign. Differences between *t*_0_ and *t*_28_ (long term). The bold values are significant at p <0.05.

## Data Availability

The data used to support the findings of this study are made available from the corresponding author upon reasonable request.

## Supplementary Materials

Supplementary Materials In accordance with established guidelines for reporting clinical trials, the CONSORT (Consolidated Standards of Reporting Trials) checklist was utilized to ensure transparency and completeness in the presentation of trial methodology and results. To check this list, please see Supplementary S1.
